# The effect of attentional load on implicit sequence learning in children and young adults

**DOI:** 10.3389/fpsyg.2014.00465

**Published:** 2014-05-21

**Authors:** Daphné Coomans, Jochen Vandenbossche, Natacha Deroost

**Affiliations:** Department of Clinical Experimental Psychology, Vrije Universiteit BrusselBrussels, Belgium

**Keywords:** implicit learning, sequence learning, dual-tasking, children, serial reaction time task

## Abstract

We investigated the effect of a secondary task on implicit sequence learning in children and young adults. A serial reaction time (SRT) task was administered to 8-to-10 year old children and 18-to-22 year old adults. Participants reacted to the location of a target presented in one of four locations on the screen with a spatially corresponding response key. Unknown to participants, the location at which the target appeared was structured according to a deterministic sequence. Occasionally, the black target dot was replaced by a red target dog. To assess the effect of attentional load on implicit sequence learning, half of the participants of each age group was assigned to the single task condition, while the other half executed the task under dual task conditions. Whereas participants in the single task condition could ignore the change in target identity, dual task participants additionally had to count the number of times the black dot was replaced by a red dog to increase the attentional load. Sequence learning was tested under single task conditions in both conditions. *Z*-transformed results indicate that young adults generally showed more sequence learning than children. Importantly, the secondary task had no effect on sequence learning in children, since children learned as much under dual task conditions as under single task conditions. Adults, on the other hand, showed a different result pattern, as they displayed more sequence learning under single task than under dual task conditions. We surmise that this result is due to the vainly attempt of adults, but not children, to integrate both sequences.

## Introduction

Many of our daily activities rely on the implicit acquisition of sequence knowledge (Clegg et al., 1998). The implicit character of this kind of knowledge is derived from the fact that sequenced information is usually learned incidentally and difficult to express (Cleeremans et al., 1998). Implicit sequence learning is mainly investigated by a serial reaction time (SRT) task, originally developed by Nissen and Bullemer (1987). In this task, a target appears in one of several locations on the screen. Participants are asked to respond to the location of this target by pressing a spatially corresponding key. Unknown to participants, the location of the target is structured according to a predetermined sequence. Learning can be assessed in two ways: (1) participants respond faster as the task progresses, denoting a training effect, and (2) participants respond significantly slower when the structured sequence is suddenly replaced by another sequence. This delay in reaction times (RTs) as a response to the omission of the structured sequence is called the sequence-specific learning effect. Because participants (1) are not informed about the presence of a sequence in the SRT task and thus acquire the sequence knowledge without intending to, and (2) are usually not able to describe the learned sequence after the experiment, the SRT task is considered a good tool to investigate implicit learning.

Implicit learning is presumed to be the default learning process and is supposed to occur independently of age and developmental level (Reber, 1993). That children can learn sequences in an implicit manner is a well-established phenomenon (Meulemans et al., 1998; Thomas and Nelson, 2001; Thomas et al., 2004; Karatekin et al., 2007; but see Weiermann and Meier, 2012). However, developmental differences in learning have been reported (Thomas et al., 2004; Janacsek et al., 2012; Weiermann and Meier, 2012; see also Savion-Lemieux et al., 2009; Ruitenberg et al., 2013 for age differences in learning in other sequence learning paradigms). For example, Thomas et al. (2004) not only found a difference in learning magnitude between children and adults, but also a differential recruitment of neural areas (like the hippocampus and parietal cortex) during sequence learning. Also other authors who have found age-related differences in learning claim that the neural areas involved in implicit sequence learning, such as the frontostriatal network, might still be maturing during childhood (Weiermann and Meier, 2012). This possibly indicates that children, in contrast to what is predicted by the age independence hypothesis (Reber, 1993), acquire sequence knowledge in a different manner than adults.

The aim of the current research was to gain more insight in the way children obtain sequence-specific knowledge by comparing learning in children and adults under single and dual task conditions. Studies on implicit sequence learning comparing the performance of children and adults have mainly used single task paradigms. This means that the sequence learning task was the only task participants had to perform, so all available attentional capacity could be devoted to the task. In contrast, in the current research we added a secondary counting task to the SRT task. Secondary tasks can be used to investigate the attentional demands of implicit learning, since the attentional capacity necessary to perform the secondary task cannot be used to learn the sequence in the SRT task. To our knowledge, the effect of a secondary task on implicit sequence learning in children has never been investigated to date. By comparing learning in children and adults under single and dual task conditions, we cannot only determine potential differences in learning between children and adults, but also the extent to which these differences are connected to different attentional demands. Though never investigated directly, there are some indications that attentional demands of sequence learning differ between age groups. More specifically, in the experiment of Thomas et al. (2004), adults showed larger parietal activity for random as compared to sequenced trials whereas this pattern was reversed in children. The authors suggested that increased activity in this region indicates larger demands on the attentional orienting system, depleting the available amount of attentional resources. Consequently, sequence learning in adults becomes less dependent on attention over learning due to increased sequence predictability as evidenced by the decreased parietal activity for sequenced trials. In contrast, the lack of a decrease in parietal activity for sequenced trials in the children suggests that sequence learning continues to be highly attention demanding because the sequence predictability remains low (Thomas et al., 2004).

However, the proposition that sequence learning in children might be more attention based than sequence learning in adults has never been investigated directly. In contrast, numerous studies have assessed the effect of a secondary (mainly tone-) counting task on sequence learning in adults (e.g., Cohen et al., 1990; Curran and Keele, 1993; Frensch et al., 1994, 1998; Heuer and Schmidtke, 1996; Hsiao and Reber, 2001). Yet, the results of these studies are somewhat equivocal. While some authors argue that implicit sequence learning operates largely independent from attentional capacity (e.g., Jiménez and Méndez, 1999), more recent studies find impaired sequence learning under dual task conditions (Shanks et al., 2005; Cohen and Poldrack, 2008; Schumacher and Schwarb, 2009; Wierzchoñ et al., 2012), at least when the secondary task is sufficiently demanding (Wierzchoñ et al., 2012). This suggests that sequence learning (in adults) has some attentional requirements.

Nonetheless, diminished attentional capacity is not the only way in which the induction of a secondary task may affect sequence learning (see Wierzchoñ et al., 2012, for an overview). One factor that may be important in the current developmental study is the extent to which participants try to integrate several streams of information. Adults tend to integrate all information they encounter. Schmidtke and Heuer (1997, see also Keele et al., 2003), for example, found a disruption of sequence learning when tones in a secondary task were randomly determined, but found enhanced learning when the two sequences were correlated. However, the urge or ability to integrate several streams of information may be age dependent, as Shin (2011) recently found that integrative learning improved with age. In her study, children were less able to integrate a response sequence (like in a typical SRT task) with a temporal sequence of RSI's than adults. Consequently, the induction of a secondary task in which stimuli are randomly varied may induce differential effects on learning in children and adults. More specifically, if adults attempt to integrate randomly varying information with the sequenced information while children do not, learning in adults might be more compromised than learning in children under dual task conditions.

In sum, dual task effects might differ between children and adults (a) because sequence learning in children is more attention-based than sequence learning in adults (in that case, we expect a larger detrimental effect of the secondary task on sequence learning in children than in adults) or (b) because adults vainly try to integrate the randomly varying stimuli from the secondary task in the SRT sequence while children do not (in which case we expect a larger effect of the secondary task on learning in adults than on learning in children).

In the current experiment, a group of 8-to-10-year old children and young adults performed an SRT task under single or dual task conditions. In the single task condition, participants performed a typical SRT task, reacting to the sequenced location of a stimulus. Under dual task conditions, participants additionally had to count the number of times the identity of the target was changed from a black dot to a red dog (this change in target identity was randomly determined). We chose to use a symbol counting task as secondary task because of two reasons. First, the task had to be rather easy to perform by children of 8-to-10 years old. Although difficulty of the secondary task was recently emphasized by Wierzchoñ et al. (2012), children might fail to perform both tasks at once if one of the tasks is too challenging. In contrast, both children and adults should be able to count symbols without much effort, which makes this task suitable to use in developmental research. Second, a symbol counting task instead of a more traditional tone counting task was used to diminish the effect of the secondary task on the timing of the sequence. In tone counting tasks, tones are presented in the inter trial interval, disrupting the timing of the sequence (Frensch et al., 1994; Stadler, 1995; Hsiao and Reber, 2001). We surmise that performing the secondary task on stimuli presented within the SRT task is less disruptive for the timing (Jiménez and Méndez, 1999).

Because most studies in adults indicate that sequence learning is affected by a secondary task, we hypothesize that the secondary task will reduce sequence learning in adults. Furthermore, if sequence learning develops differently in children than in adults and learning in children is more attention-based, then the secondary task will affect learning in children even more than learning in adults. However, if adult participants are in vain trying to integrate both streams of information (i.e., the location sequence and the randomly varying stimulus identity) whereas children are not, dual task learning is expected to be more interrupted in adults than in children.

## Materials and methods

### Ethics statement

The study was approved by the Medical Ethics Committee of the Vrije Universiteit Brussel (reference 2013/046). An informed consent was obtained from all subjects. For young adults, this informed consent was obtained from themselves. In case of the children, the informed consent was obtained from their parents.

### Participants

Thirty-nine young adults executed the study in return for course credit of an introductory psychology course. Twenty participants (5 male, mean age = 18.95, *SD* = 1.28) performed the experiment in the single task condition, 19 participants (5 male, mean age = 19.42, *SD* = 1.39) performed the experiment in the dual task condition. In addition, 39 8-to-10 year old children participated in the study. This age group was chosen because 8-to-10 year old children are capable of counting signals without much effort. Nineteen children (6 male, mean age = 9.01, *SD* = 0.68) performed the experiment in the single task condition, and 20 children (6 male, mean age = 9.14, *SD* = 0.58) performed the experiment in the dual task condition. After the experiment, the children received a small present to thank them for their participation.

Participants in the single and dual task condition were matched for age [*t*_(37)_ = 0.64, *p* = 0.523 for the children and *t*_(37)_ = 1.10, *p* = 0.277 for the adults] and sex [χ^2^_(1, *N* = 39)_ = 0.01, *p* = 0.915 for the children and χ^2^_(1, *N* = 39)_ = 0.01, *p* = 0.925 for the adults].

### Stimuli and apparatus

The experiment was programmed and run in the software program E-Prime 2 Professional (Schneider et al., 2002). Adult participants executed the experiment on Intel Core I3 personal computers with 17-inch LCD monitors individually in semi-darkened cubicles of the psychological laboratory of the Vrije Universiteit Brussel. The children performed the experiment individually in a quiet room in their school on an Intel Core 2 Duo personal computer with 15.6-inch monitor.

In both the single and the dual task condition, 4 white rectangles, measuring 2 cm width and 1.8 cm height, were presented horizontally on a gray background. The white rectangles were separated by 2.5 cm or 2.86° visual angle from a viewing distance of 50 cm. On every trial, a target appeared in one of the white rectangles. This target consisted of a black dot (Webdings point size 12), with a diameter of 1 cm, or a red dog (Webdings point size 26), measuring 1.3 cm width and 1.2 cm height. A red dog was used because this would attract more attention than a red dot, reducing the chances that the signal would be overlooked (and hence remain uncounted) by the children.

### Procedure

Participants were asked to respond as quickly and accurately as possible to the target location with a spatially corresponding response key. When the target appeared at the leftmost location, the “C” key of an AZERTY-keyboard had to be pressed with the left middle finger. Additionally, the left location required a “V” key response with the left index finger, the right location a “B” key response with the right index finger and the rightmost location an “N” key response with the right middle finger. In case of an erroneous response an error message was displayed in Dutch for 750 ms. After a response-stimulus interval of 250 ms the next trial was presented.

The experiment started with two practice blocks of 50 trials. In these practice blocks, the location of the target changed randomly. In the first practice block, the target was always a black dot. In the second practice block, the black dot was replaced by a red dog on 10 of the 50 trials. The trials on which the black dot was replaced by a red dog were randomly determined, with the exception that the target could never be a red dog on the first trial of the block or on 2 consecutive trials. In the single task condition, participants were told to ignore the identity of the target and to keep reacting as fast as possible to its location. In the dual task condition, on the other hand, participants were asked to count the number of times the black dot was replaced by a red dog in addition to reacting as fast as possible to the target's location. After this block, participants in the dual task condition were asked to indicate the number of times the target had been replaced. After each block, participants received feedback about their error rates and RTs during the last block, followed by a rest break of 30 s. They received no feedback about the counting task.

After practice, participants completed 8 training blocks of 72 trials. In these blocks, the target was replaced by a red dog in 15–20 trials or 20.83–27.78% of the trials. Participants in the single task condition could ignore the replacement of the black dot by the red dog, while participants in the dual task condition had to keep track of the number of times this happened. These latter participants had to indicate the number of times the target had changed identity after each block.

Unknown to participants, the target location in these training blocks was structured according to an 8-element deterministic sequence. For half of the participants, the sequence was 42132431 (S1, with 1 referring to the leftmost location, 2 to the left location, 3 to the right location, and 4 to the rightmost location). For the other participants, the reversed sequence was used, i.e., 13423124 (S2). To avoid that participants would learn the sequence explicitly, the sequence started at different locations in the different training blocks.

After the training phase, three test blocks of 72 trials were administered (Blocks 9–11). These test blocks were performed under single task conditions for all participants as the target only consisted of a black dot. In the 9th and the 11th block, the same sequence as in the training phase was inserted on the target's location. In Block 10, the reversed sequence was used to assess sequence learning. More specifically, participants trained with S1 encountered S2 in Block 10, and vice versa.

Finally, awareness of the sequence was assessed by a structured interview in which both judgment and structural knowledge was determined (Dienes and Scott, 2005; see also Verwey and Abrahamse, 2012). The first two questions assessed judgment knowledge, or the knowing that one had sequence knowledge. Participants were asked whether they noticed anything about the experiment (Question 1) and whether they had noticed that the location of the target had been structured in the experiment (Question 2). Next, structural knowledge, or knowledge about the nature of the sequence, was determined. To assess this kind of knowledge, participants were asked to describe the sequence they encountered during the task (Question 3). In doing so, they could place their fingers on the keyboard and show the movements they believed had appeared during the experiment.

## Results

### Counting task

First, we assessed whether participants in the dual task condition were sufficiently accurate when counting the number of times the identity of the target was changed. Therefore, we calculated the percentage of errors participants made over all blocks per group (children vs. adults). If this percentage exceeded the mean error rate percentage plus two standard deviations, the participant was excluded from further analyses. As a consequence, one male participant in the children's group and one male participant in the adults' group were excluded. The remaining children made 5.31% (*SD* = 4.50) counting errors, the remaining adults had a mean counting error rate of 4.12% (*SD* = 3.24). One child and 1 adult performed the secondary task perfectly. There was no difference between the performance of children and the performance of adults on the counting task, *t*_(35)_ = 0.91, *p* = 0.37 (see the Supplemantary Table for an overview of the error rate percentage per block per participant).

Additionally, to test whether participants truly updated their count after the presentation of a red dog instead of merely estimating the number of red dogs after each block, we compared median RTs on regular (“black dot”) trials with median RTs on irregular (“red dog”) trials for children and adults separately. A mixed ANOVA was computed with Trial type (regular vs. irregular) and Block (Blocks 1–8—only regular trials were presented in the test Blocks 9–11) as within-subjects factors and Condition (single vs. dual) as between-subjects factor. Erroneous responses and responses after an error were excluded from the analyses. When the sphericity assumption was not fulfilled, the Greenhouse-Geisser correction is reported. A significance level of 0.05 was used.

#### Children

**Figure 1:**
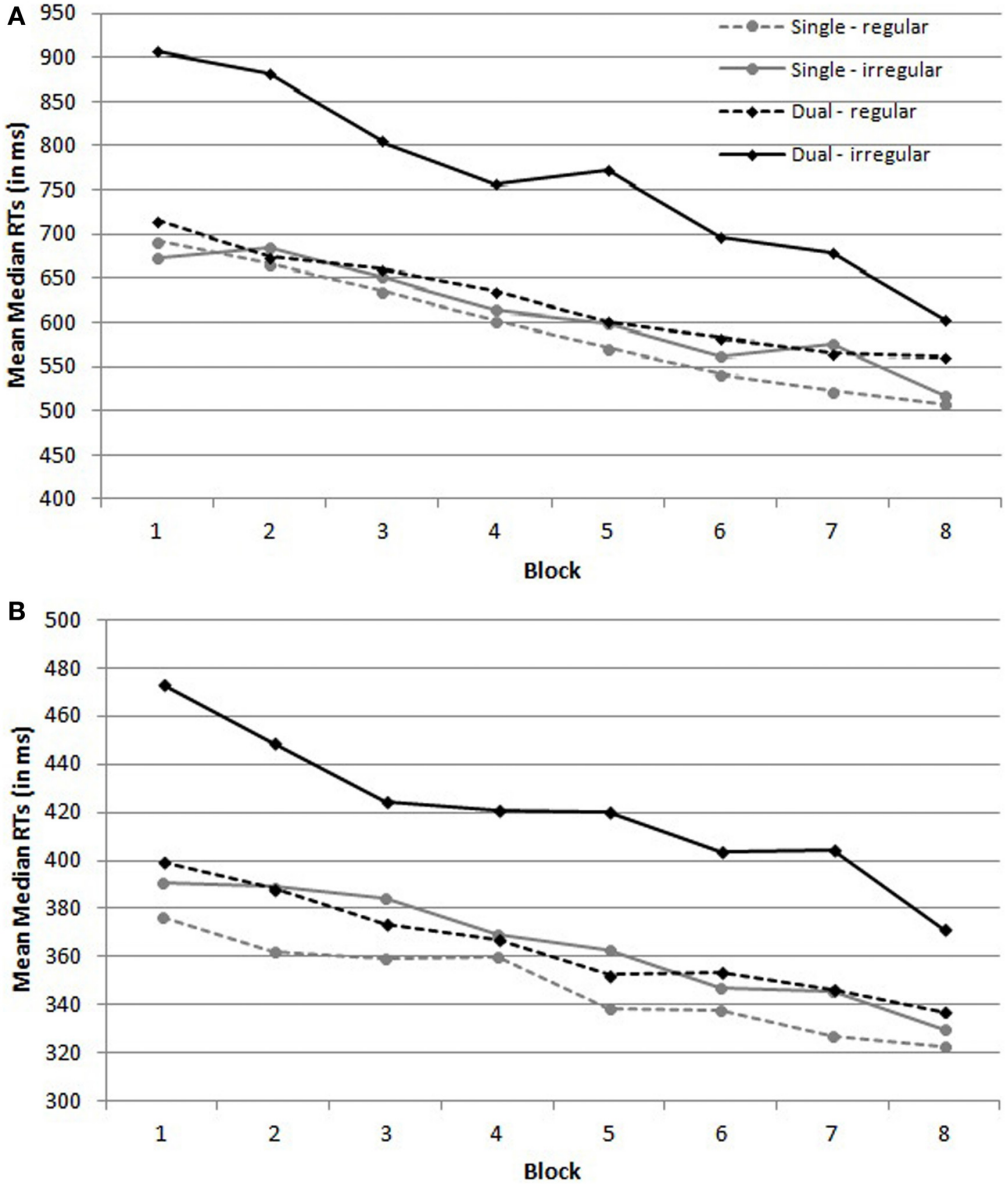
**Mean median reaction times (RTs) on regular and irregular trials per condition of the (A) children and (B) adults**. Regular trials are the trials on which a black dot was presented as a target; irregular trials are the trials on which a red dog was presented as a target.

Figure 1A depicts the mean median RTs on regular and irregular trials of the children per condition. The ANOVA revealed no main effect of Condition, *F*_(1,36)_ = 2.34, *MSE* = 553,898, *p* = 0.13, η^2^_*p*_ = 0.06, indicating that mean RTs were comparable in the single and the dual task condition. Importantly, there was a main effect of Trial type, *F*_(1,36)_ = 14.20, *MSE* = 64,825, *p* < 0.001, η^2^_*p*_ = 0.28 and a Trial type by Condition interaction, *F*_(1,36)_ = 8.58, *MSE* = 64,825, *p* = 0.0059, η^2^_*p*_ = 0.19. To investigate this interaction, planned contrasts were computed. These indicated that there was no effect of Trial Type in the single task condition, *F* < 1, but that participants were slower on irregular trials than on regular trials in the dual task condition, *F*_(1,36)_ = 22.42, *MSE* = 64,825, *p* < 0.001. Furthermore, there was a main effect of Block, *F*_(3,102)_ = 32.85, *MSE* = 28,266, *p* < 0.001, η^2^_*p*_ = 0.48, indicating that RTs decreased over blocks, but there was no Block by Condition interaction, *F* < 1. Finally, there was a Trial type by Block interaction, *F*_(3, 123)_ = 3.03, *MSE* = 8796, *p* = 0.027, η^2^_*p*_ = 0.08 and a Trial type × Block × Condition interaction, *F*_(3, 123)_ = 4.05, *MSE* = 8796, *p* = 0.0064, η^2^_*p*_ = 0.10. These latter interactions suggest that the decrease in RTs over Blocks was larger for irregular trials in the dual task condition than for the other conditions (see Figure 1A).

#### Adults

Figure 1B depicts the mean median RTs on regular and irregular trials of the adults per condition. The same ANOVA on adult's RTs demonstrated a main effect of Condition, *F*_(1,36)_ = 5.11, *MSE* = 39,313, *p* = 0.030, η^2^_*p*_ = 0.12, suggesting that RTs were higher under dual than under single task conditions. Importantly, there was a main effect of Trial type, *F*_(1,36)_ = 133.32, *MSE* = 1513, *p* < 0.001, η^2^_*p*_ = 0.79, and a Trial type by Condition interaction, *F*_(1,36)_ = 38.27, *MSE* = 1513, *p* < 0.001, η^2^_*p*_ = 0.52. Although participants in the dual task condition were more slowed by irregular trials than participants in the single task condition, planned contrasts showed that participants in both conditions were significantly slower on irregular than on regular trials, *F*_(1,36)_ = 15.16, *MSE* = 1513, *p* < 0.001 in the single task condition and *F*_(1,36)_ = 149.37, *MSE* = 1513, *p* < 0.001 in the dual task condition. The main analysis also indicated that RTs decreased over blocks, *F*_(4,158)_ = 38.46, *MSE* = 1644, *p* < 0.001, η^2^_*p*_ = 0.52, but there was no Block × Condition interaction, *F*_(4,158)_ = 1.34, *MSE* = 1644, *p* = 0.25, η^2^_*p*_ = 0.04. Finally, there was a Trial type by Block interaction, *F*_(4,161)_ = 3.76, *MSE* = 600, *p* = 0.004, η^2^_*p*_ = 0.10, reflecting the larger decrease of RTs on irregular trials than on regular trials over blocks, but no Trial type × Block × Condition interaction, *F*_(4,161)_ = 1.43, *MSE* = 600, *p* = 0.22, η^2^_*p*_ = 0.04.

To summarize, both children and adults responded slower on irregular than on regular trials under dual task conditions. Moreover, adults also responded slower on irregular trials under single task conditions, yet this delay was not as large as under dual task conditions. All together, we can conclude that participants paid attention to the target identity, probably updating their count before responding to the SRT trial.

### SRT task

Again, only correct responses were included in the RT analyses, and responses after an erroneous response were also excluded from the analyses. While responding to the location of the target, children made 3.88% errors (*SD* = 2.01) in the single task condition and 4.13% (*SD* = 2.95) in the dual task condition. Adults made 2.30% errors (*SD* = 1.48) in the single task condition and 1.82% (*SD* = 1.34) in the dual task condition.

Because the children generally responded slower and made more errors than the adults, which could affect the size of the learning effect, we *z*-transformed the median RTs and error rates with reference to the participant's global response time or error rate^1^. The further reported analyses were all performed on the median *z*-transformed RTs and *z*-transformed error rates per block.

#### Training effect

A training effect refers to the enhancement of performance over training and may result from both generalized learning and sequence-specific learning. To assess this effect, we calculated a mixed ANOVA with Training (training Blocks 1–8) as within-subjects factor and Condition (single vs. dual) and Group (children vs. adults) as between-subjects factors.

**Figure 2:**
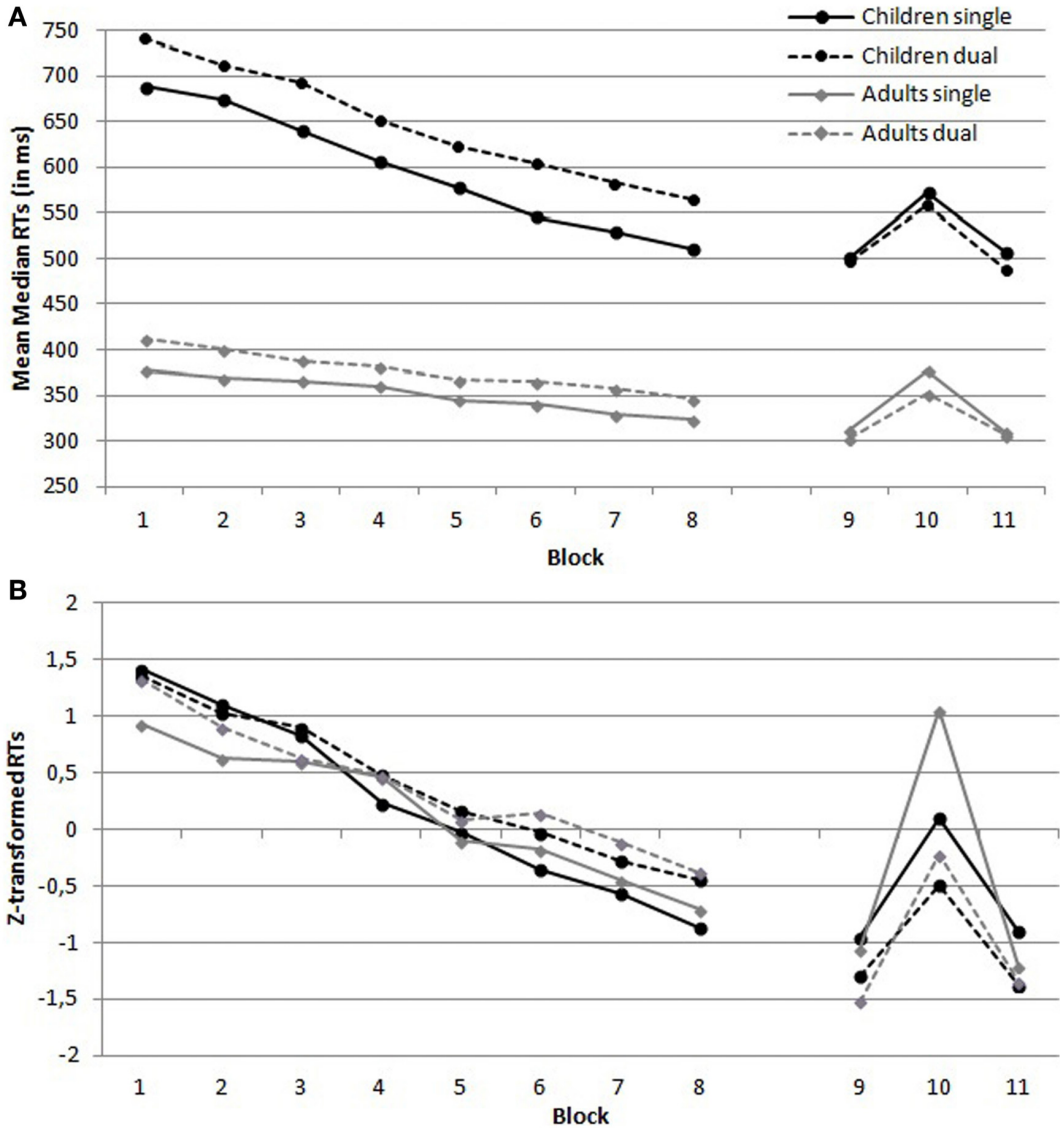
**(A)** Untransformed and **(B)** *z*-transformed mean median reaction times (RTs) per block in function of Group (children vs. adults) and Condition (single vs. dual task condition).

***Reaction times.*** Figure 2 provides an overview of the untransformed (Figure 2A) and transformed (Figure 2B) mean median RTs per block in function of the four conditions. The analysis on *z*-transformed data demonstrated a main effect of Condition, which suggested that RTs were higher under dual task conditions than under single task conditions, *F*_(1, 72)_ = 74.41, *MSE* = 0.086, *p* < 0.001, η^2^_*p*_ = 0.51. There was no main effect of Group, *F*_(1, 72)_ = 2.93, *MSE* = 0.086, *p* = 0.091, η^2^_*p*_ = 0.04. In addition, there was no Condition × Group interaction, *F*_(1, 72)_ = 1.67, *MSE* = 0.086, *p* = 0.20, η^2^_*p*_ = 0.02. Furthermore, the analysis demonstrated a main effect of Training, which suggests that RTs decreased over training, *F*_(7, 504)_ = 104.89, *MSE* = 0.31, *p* < 0.001, η^2^_*p*_ = 0.59. The decrease in RTs was comparable in both conditions, as there was no Training by Condition interaction, *F* < 1. There was, however, a Training by Group interaction, *F*_(7, 504)_ = 2.53, *MSE* = 0.31, *p* = 0.015, η^2^_*p*_ = 0.03. This reflects the larger training effect in children as compared to adults. Finally, there was no three-way interaction, *F* < 1.

***Error rates.*** The same analysis on *z*-transformed error rates did not yield any significant results (all *p* > 0.24), except a main effect of Group, *F*_(1, 72)_ = 3.99, *MSE* = 0.34, *p* = 0.050, η^2^_*p*_ = 0.05, with children generally showing more errors than adults.

#### Sequence-specific learning

Next, we determined whether participants had acquired sequence-specific knowledge and whether this amount of knowledge differed between groups and conditions. Sequence-specific knowledge was determined in the three test blocks, by comparing the performance in the trained sequence blocks (average of Blocks 9 and 11) with the performance in the block where the sequence was reversed (Block 10). If participants had acquired sequence-specific knowledge, their performance should decline when the trained sequence was omitted in Block 10. A 2 (Sequence learning: average of Blocks 9 and 11 vs. Block 10) × 2 (Condition: single vs. dual) × 2 (Group: children vs. adults) mixed ANOVA was performed.

**Figure 3:**
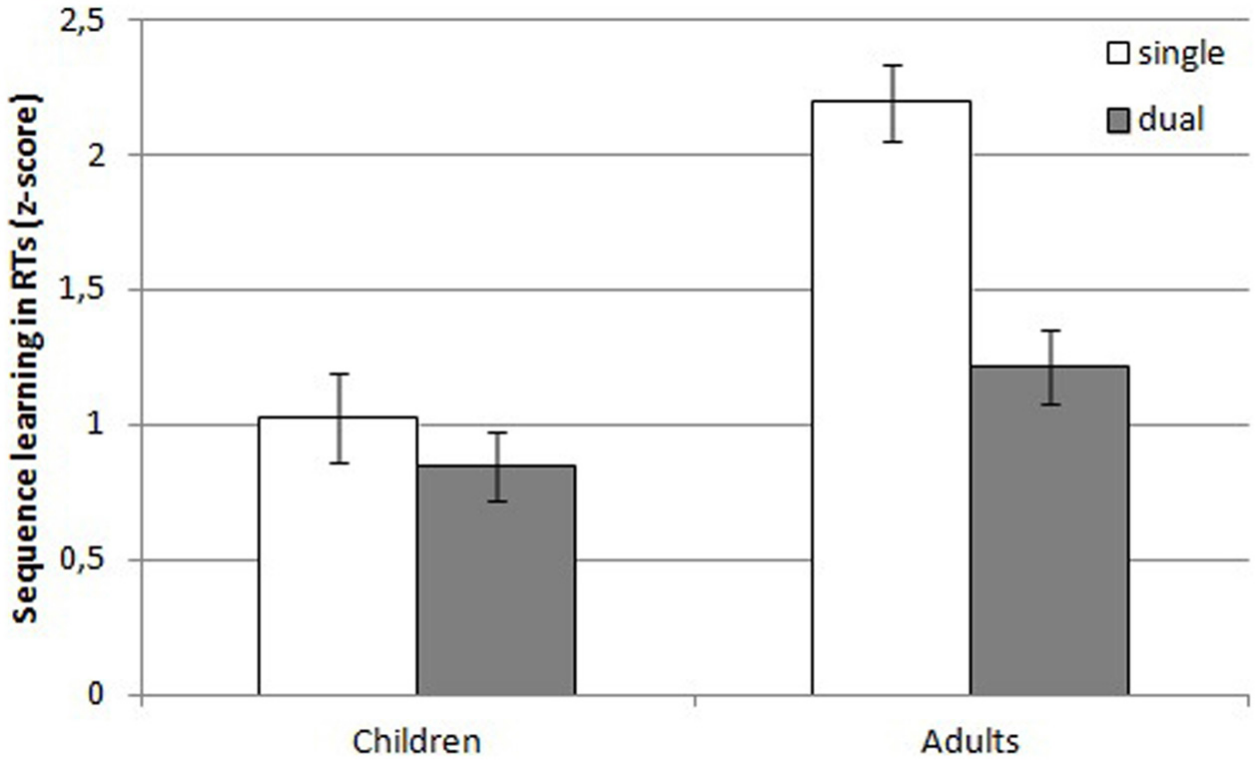
***Z*-transformed learning effects in reaction times of children and adults in the single and dual task condition**.Error bars denote standard errors of the mean.

***Reaction times.*** An illustration of the *z*-transformed learning effects in children and adults for both conditions can be found in Figure 3. There was a main effect of Condition, *F*_(1, 72)_ = 71.35, *MSE* = 0.22, *p* < 0.001, η^2^_*p*_ = 0.50 and Group, *F*_(1, 72)_ = 9.60, *MSE* = 0.22, *p* = 0.0028, η^2^_*p*_ = 0.12. *Z*-transformed RTs in the single task condition were higher than in the dual task condition and RTs of the adults were higher than RTs of the children^2^. The Condition by Group interaction, though, failed to reach significance, *F*_(1, 72)_ = 3.80, *MSE* = 0.22, *p* = 0.055, η^2^_*p*_ = 0.05. There was a main effect of Sequence learning, *F*_(1, 72)_ = 332.12, *MSE* = 0.20, *p* < 0.001, η^2^_*p*_ = 0.82, suggesting that participants learned the sequence. However, this learning effect was modulated by Condition, as indicated by a Sequence learning × Condition interaction, *F*_(1, 72)_ = 15.99, *MSE* = 0.20, *p* < 0.001, η^2^_*p*_ = 0.18. More sequence learning was observed under single than under dual task conditions. Moreover, sequence learning was also influenced by Group, as demonstrated by a Sequence learning by Group interaction, *F*_(1, 72)_ = 27.96, *MSE* = 0.20, *p* < 0.001, η^2^_*p*_ = 0.28. Adults showed more sequence learning than children. Finally, the three-way Sequence learning × Condition × Group interaction suggested that sequence learning in adults was more affected by condition than sequence learning in children, *F*_(1, 72)_ = 7.63, *MSE* = 0.20, *p* = 0.007, η^2^_*p*_ = 0.10.

Next, planned comparisons were computed to assess whether learning in children and adults differed between the conditions (see Table 1 for an overview of the transformed and untransformed learning effects). These indicated that learning in children was similar under single and dual task conditions, *F* < 1. Learning in adults, on the other hand, was significantly different under single and dual task conditions, indicating that more learning occurred under single than under dual task conditions, *F*_(1, 72)_ = 22.82, *MSE* = 0.20, *p* < 0.001.

**Table 1:** **Untransformed and transformed learning effects with their standard deviation (*SD*) for all conditions**.

**Group**	**Condition**	**RTs**		**Error rates**	
		**Untransformed**	***Z*-transformed**	**Untransformed**	***Z*-transformed**
Children	Single	69 ms (43.37)	1.03 (0.74)^***^	0.63 (1.86)	0.52 (1.08)^*^
	Dual	67 ms (35.96)	0.85 (0.57)^***^	1.21 (1.70)	0.72 (1.01)^**^
Adults	Single	67 ms (19.51)	2.19 (0.14)^***^	0.98 (1.89)	0.68 (1.28)^*^
	Dual	47 ms (23.10)	1.21 (0.59)^***^	1.39 (1.96)	1.22 (1.65)^**^

Learning effects were calculated by extracting the performance in trained test Blocks 9 and 11 from the performance in untrained Block 10. Z-transformed learning effects were analyzed by comparing them to 0 with a one sample t-test.

* Significant at the 0.05 level,

** Significant at the 0.01 level,

*** Significant at the 0.001 level.

In sum, adults showed more sequence-specific learning in general than children. Moreover, only adults showed more learning under single than under dual task conditions. Importantly, though, all groups showed a learning effect that differed significantly from 0, as indicated by a one sample *t*-test (see Table 1).

***Error rates.*** A 2 (Sequence learning) × 2 (Condition) × 2 (Group) ANOVA demonstrated a main effect of Group, *F*_(1, 72)_ = 4.55, *MSE* = 0.79, *p* = 0.036, η^2^_*p*_ = 0.06, suggesting that adults made on average more errors than children. There was also a significant main effect of Sequence learning, indicating that participants made more errors in untrained Block 10 than in trained Blocks 9 and 11, *F*_(1, 72)_ = 28.73, *MSE* = 0.81, *p* < 0.001, η^2^_*p*_ = 0.29. No other effects were significant (all *p* > 0.21).

An overview of the untransformed and transformed learning effects in error rates can be found in Table 1. All learning effects were significant.

#### Explicit knowledge

Finally, we assessed whether participants acquired explicit knowledge about the sequence during the SRT task. The results of the three questions of the structured interview can be found in Table 2.

**Table 2:** **Explicit knowledge**.

**Group**	**Condition**	**Notice anything? (Q1)**	**Notice sequence? (Q2)**	**Sequence reproduction(Q3)**	
		**%**	**%**	**% (with SD)**	**Range**
Children	Single task	0.00	0.00	7.89 (11.94)	0–2
	Dual task	0.00	0.00	6.58 (14.05)	0–4
Adults	Single task	50.00	55.00	18.13 (23.81)	0–5
	Dual task	22.22	33.33	6.25 (15.01)	0–4

Results of the structured interview administered after the SRT task. Participants were scored positively on the first question (Q1) when they mentioned a pattern or repetition in the task. Participants were scored positively on the second question (Q2) when they responded with “yes” to the question whether they had noticed that the location of the target had been structured in the experiment. The percentage of the sequence participants were able to generate is displayed in the fifth column (Q3). This percentage was derived from the longest correct sequence the participant was able to reproduce. In the sixth column, the range of the number of elements [lowest – highest] participants could reproduce was presented. A reproduction of 8 elements reflects a perfect reproduction (though none of the participants reached this number).

***Judgment knowledge.*** First, we assessed whether participants differed in the extent to which they had judgment knowledge. With respect to the first question (“Did you notice anything about the experiment?”), the difference between the age groups was significant in both the single and the dual task condition, χ^2^_(1, *N* = 39)_ = 12.78, *p* < 0.001 and χ^2^_(1, *N* = 37)_ = 4.73, *p* = 0.030, respectively. Consequently, in both the single and the dual task condition, adults were more prone to notice a sequence in the task than children. In contrast, the difference between the single and the dual task condition failed to reach significance in the adult's group, χ^2^_(1, *N* = 38)_ = 3.14, *p* = 0.076 (this latter analysis was not performed on the children's data because none of them noticed a sequence in the task, see Table 2).

The second question examined whether participants had noticed that the location of the target had been structured in the experiment. More adult than child participants noticed a sequence in the task, in both the single and the dual task condition, χ^2^_(1, *N* = 39)_ = 14.56, *p* < 0.001 and χ^2^_(1, *N* = 37)_ = 7.56, *p* = 0.006. In the adult's group, the difference between the single and dual task condition was non-significant, χ^2^_(1, *N* = 38)_ = 1.80, *p* = 0.18.

Explicit knowledge about a deterministic sequence is known to increase the sequence learning effect (e.g., Destrebecqz, 2004; Stefaniak et al., 2008). In order to determine whether the larger sequence learning effect under single than under dual task conditions in adults could be explained by more judgment knowledge, we assessed whether sequence learning differed between adults that showed judgment knowledge and adults that did not (again, this analysis was not performed on the children's data since none of them demonstrated judgment knowledge). To this end, we performed a univariate ANOVA on (*z*-transformed) sequence learning with Condition (single vs. dual) and Judgment knowledge expressed in Question 1 (yes vs. no) as between-subjects factors. A main effect of Condition indicated that, as expected, there was more learning under single than under dual task conditions, *F*_(1, 34)_ = 20.34, *MSE* = 0.33, *p* < 0.001, η^2^_*p*_ = 0.37. There was no main effect of Judgment knowledge, *F*_(1, 34)_ = 1.09, *MSE* = 0.33, *p* = 0.30, η^2^_*p*_ = 0.03, and the Condition by Judgment knowledge interaction failed to reach significance, *F*_(1, 34)_ = 3.11, *MSE* = 0.33, *p* = 0.087, η^2^_*p*_ = 0.084. Surprisingly, although not significant, this tendency is opposite to what would be expected if the larger learning effect under single task conditions would be evoked by explicit knowledge, since participants under single task conditions without judgment knowledge displayed more sequence learning (*M* = 2.48, *SD* = 0.55) than participants with judgment knowledge (*M* = 1.90, *SD* = 0.55). Sequence learning effects under dual task conditions seem rather similar in participants with and without judgment knowledge (*M* = 1.33, *SD* = 0.46 vs. *M* = 1.18, *SD* = 0.63). Consequently, this analysis does not support the assumption that the difference in learning in adults between the single and the dual task condition is due to explicit knowledge.

***Structural knowledge.*** We measured structural knowledge by asking participants to describe the sequence (see Table 2, question 3). A univariate ANOVA with Condition (single vs. dual task) and Group (children vs. adults) as between subjects factors and the percentage of correct reproduced elements as dependent variable demonstrated that the main effect of Condition failed to reach significance, *F*_(1, 72)_ = 3.50, *MSE* = 284, *p* = 0.065, η^2^_*p*_ = 0.05. There was no main effect of Group, *F*_(1, 72)_ = 2.09, *MSE* = 284, *p* = 0.152, η^2^_*p*_ = 0.03, so the amount of structural knowledge did not differ between children and adults. There was also no Condition by Group interaction, *F*_(1, 72)_ = 1.85, *MSE* = 284, *p* = 0.178, η^2^_*p*_ = 0.03.

Next, we assessed whether structural knowledge correlated with (*z*-transformed) sequence learning. This analysis revealed that in none of the groups, a significant correlation between sequence learning and structural knowledge was present (children single: *r* = 0.06, *p* = 0.81; children dual: *r* = 0.02, *p* = 0.93; adults single: *r* = 0.08, *p* = 0.72; adults dual *r* = 0.27, *p* = 0.28).

Finally, to make sure that our results were not influenced by explicit knowledge, we reran our main analysis with the exclusion of participants that were able to reproduce half of the sequence (so, 4 out of 8 elements). This resulted in the omission of 1 child in the dual task condition, 5 adults in the single task condition (of which 1 could reproduce 5 elements), and 1 adult in the dual task condition. The 2 (Sequence learning) × 2 (Condition: single vs. dual) × 2 (Group: children vs. adults) mixed ANOVA revealed that the results remained the same without these “explicit” participants. There was a main effect of Condition, Group and a Condition by Group interaction, *F*_(1, 65)_ = 69.43, *MSE* = 0.23, *p* < 0.001, η^2^_*p*_ = 0.52; *F*_(1, 65)_ = 8.26, *MSE* = 0.23, *p* = 0.0055, η^2^_*p*_ = 0.11; and *F*_(1, 65)_ = 4.21, *MSE* = 0.23, *p* = 0.044, η^2^_*p*_ = 0.06. Importantly, there was still a main effect of Sequence learning, a Sequence learning × Condition and a Sequence learning × Group interaction, *F*_(1, 65)_ = 276.57, *MSE* = 0.21, *p* < 0.001, η^2^_*p*_ = 0.81; *F*_(1, 65)_ = 14.90, *MSE* = 0.21, *p* < 0.001, η^2^_*p*_ = 0.19; and *F*_(1, 65)_ = 23.43, *MSE* = 0.21, *p* < 0.001, η^2^_*p*_ = 0.26, respectively. The three-way Sequence learning by Condition by Group interaction remained significant too, *F*_(1, 65)_ = 6.92, *MSE* = 0.21, *p* = 0.011, η^2^_*p*_ = 0.10. Consequently, even with the exclusion of participants that showed some explicit knowledge after the experiment, adults showed more sequence knowledge than children and, more importantly, more sequence knowledge was observed under single than under dual task conditions in adults.

## Discussion

In the current study, we investigated whether potential age related differences in sequence learning might be attributable to differential attentional demands in children and adults. Therefore, we compared the effect of a secondary counting task on sequence learning in 8-to-10 year old children and young adults. Participants in the single task condition performed a standard SRT task, while participants in the dual task condition additionally had to keep track of the number of times the target identity changed from a black dot to a red dog. Adults showed more sequence learning under single than under dual task conditions. In contrast, children showed equal sequence learning in both conditions. In addition, adults showed overall more sequence learning than children.

Importantly, though, we should take into account that these conclusions were drawn based on *z*-transformed data. A *z*-transformation is, however, affected by the variability in the data, which in fact may contribute to learning itself (see also Janacsek et al., 2012). Because the children in the current experiment showed a larger decline in RTs in the training blocks than the adults, their variability (and thus standard deviation) was larger, leading to smaller *z*-transformed learning effects and possibly an underestimation of the children's learning effects. When looking at the raw instead of the transformed data, another sequence learning pattern emerges (see Table 1). Children under single and dual task conditions and adults under single task conditions showed an equal learning effect of 67 ms (children dual and adults single) to 69 ms (children single). Only adults under dual task conditions showed less learning, namely 47 ms. Consequently, if we would only take the raw data into account, we would have to conclude that children show as much learning as adults, and that adults, but not children, show impaired learning under dual task conditions. Possibly, this result may be explained by assuming that child learning in the current experiment was at ceiling. In other words, children already showed a very large sequence learning effect under dual task conditions, so they were possibly unable to display an even larger learning effect under single task conditions. Therefore, the impact of the dual task may not have been visible in the RTs of the children. Adults, on the other hand, did not show an enormous learning effect under dual task conditions, so they were able to show a larger learning effect under single task conditions. However, better learning in children is in disagreement with research showing either equal (e.g., Meulemans et al., 1998) or reduced learning in children (e.g., Thomas et al., 2004; Weiermann and Meier, 2012). Moreover, analysing raw data when a clear baseline difference is present may lead to false conclusions, as the effect of the manipulation is likely overestimated in the slowest group (Faust et al., 1999). In the current research, this would imply that the learning effect of the children would be overestimated compared to that of the adults. Dealing with baseline differences is a major challenge in developmental research, and so far no clear consensus has been reached. Following other developmental studies, we chose to *z*-transform the data with reference to the participant's own performance to overcome this problem and to allow comparison between children and adults (e.g., Thomas et al., 2004; Janacsek et al., 2012, see also Faust et al., 1999). Nevertheless, taking into account the above-stated concerns with data transformations, we believe that especially the result that children showed overall less learning than adults should be carefully interpreted.

Since there are no obvious differences between our study and previous SRT studies that failed to expose a difference in learning between children and adults (e.g., Meulemans et al., 1998; Karatekin et al., 2007), it is not entirely clear why adults showed overall better sequence learning than children in the current study (in the transformed data). Thomas et al. (2004), who demonstrated a difference in learning magnitude between 7-to-10 year old children and adults, assumed that sequence complexity might account for the different results. More specifically, they proposed that children might have more difficulties with learning complex sequences than adults, whereas simple sequences would not elicit age differences. However, we used an 8-element deterministic sequence that was likely less complex than the 10-element deterministic sequences used in most previous studies. So, at present, an explanation for the observed difference is lacking. Future research is necessary to determine what factors may contribute to age-related differences in sequence learning, among which the effect of the data transformation.

A more interesting result of the current experiment is that learning in adults was influenced by the secondary task, while learning in children was not^3^. This indicates that, although both groups devoted attention to the secondary task as their responses were slowed on trials in which the target identity changed, this did not affect learning in children. Consequently, sequence learning in children does not seem to be more attention dependent than sequence learning in adults. So, why was learning in adults and not in children affected by the presence of a secondary task? It does not seem very plausible that learning in adults is more attention dependent than learning in children (see Thomas et al., 2004). We surmise that adults, but not children, learned on multiple levels. For example, adults may have tried to acquire a perceptual sequence by predicting when the identity of the target would be changed, or to integrate both the perceptual (target identity) and the response (location) sequence. Previous research has shown that adults can learn a perceptual sequence (e.g., Mayr, 1996; Remillard, 2003; Deroost and Soetens, 2006; Coomans et al., 2012). In addition, adults also show integration of several streams of information (e.g., Schmidtke and Heuer, 1997). However, research with children on this matter is scarce. To our knowledge, it remains to be determined whether children can learn a perceptual sequence in the SRT task. Moreover, as we already mentioned in the introduction, Shin (2011) found that integrative learning improved with age. Consequently, adults may have tried in vain to learn or integrate the perceptual (target identity) sequence with the response (location) sequence, whereas children did not. This probably only happened under dual task conditions, as previous research demonstrated that perceptual sequences are only learned if attention is paid to the perceptual stimulus (e.g., Jiménez and Méndez, 1999). Consequently, learning in adults might have been compromised under dual task conditions because adults were trying to integrate randomly varying information. Learning in children, on the other hand, was not disturbed because the children only learned the location sequence, without attempting to integrate the other (random) sequence.

This latter hypothesis can be translated to the theoretical model of Keele et al. (2003) by stating that the multidimensional system might not function to its full capacity until adulthood (Shin, 2011). According to the model of Keele et al. (2003), sequences can be learned by two systems. The unidimensional system forms associations between successive stimuli that are presented within one single dimension, while the multidimensional system supports integrated learning over different dimensions. Under single task conditions, learning is supported by the two systems. However, because the multidimensional system tries to associate all the attended information in one representation, learning in the multidimensional system will be compromised under dual task conditions if random information is provided. This might have been the case for the adult participants in the current study, who also attended the randomly varying stimulus identity and tried to integrate this information in the response (location) sequence. We surmise that the children in our study were mainly relying on the unidimensional system under both single and dual task conditions because brain areas involved in multidimensional learning, like areas within the frontal and temporal lobes, are not fully developed yet (Giedd et al., 1999; Shin, 2011). Thus, we hypothesize that adults rely on both the uni- and multidimensional system to acquire sequence knowledge, whereas children mainly rely on the unidimensional system (Keele et al., 2003).

Importantly, according to Keele et al. (2003), both learning systems acquire sequence knowledge without the need for attentional capacity, as associations between sequenced events are formed automatically. Consequently, if our assumptions about the use of these systems in children and adults are correct, learning in both the children and adults would not necessarily have to depend on attentional capacity. However, these conclusions are merely speculative at present so more research is necessary to support them.

Integrative learning is not the only way through which sequence learning might be compromised when a secondary task is performed. For example, Schumacher and Schwarb (2009) argue that the overlap of central processes like response selection is responsible for disrupted sequence learning under dual task conditions. Additionally, and possibly important for the current study, Jiménez and Vázquez (2005) stress that the development of explicit knowledge might account for diminished sequence learning under dual task conditions. These latter authors found that learning of a deterministic sequence was more affected by a secondary task than learning of a probabilistic sequence, which is known to be less prone to elicit explicit knowledge. That adults developed explicit knowledge, especially under single task conditions, while children continued to rely on implicit knowledge in both conditions (e.g., Karatekin et al., 2007) would be an obvious explanation for the result that adults were more affected by the secondary task than children. However, the analyses of explicit knowledge in our experiment do not support this explanation. If the larger sequence learning effect in adults under single task conditions was due to explicit knowledge, we would expect larger learning effects in participants that showed explicit knowledge as opposed to participants who did not show any explicit knowledge after the experiment. Yet, although adults displayed more judgment knowledge than children, the sequence learning effect in adults was unaffected by judgment knowledge. In addition, structural knowledge did not differ significantly between children and adults. Moreover, our results remained the same when participants with explicit structural knowledge were excluded from the analysis. Thus, this kind of explicit knowledge also unlikely explains the differential effect of the secondary task on learning in children and adults. Nevertheless, it is possible that the used method, i.e., a structured interview, was not sensitive enough to capture all explicit knowledge. Future research could examine whether explicit knowledge was responsible for the current results by using a probabilistic sequence, which is known to prevent sequence awareness, instead of a deterministic sequence and a more sensitive *post-hoc* awareness measure such as a process dissociation procedure (Destrebecqz and Cleeremans, 2001). Because previous research has shown that children can already control their sequence knowledge at the age of two (Bremner et al., 2007), this task may provide useful information in the future.

To conclude, we found that adults show less sequence learning when they have to perform a secondary task in addition to an SRT task as compared to when they perform the SRT task under single task conditions. In contrast, children's learning in an SRT task is not affected by a secondary task. Although we cannot exclude the possibility that explicit knowledge influenced our results, we did not find any support for this explanation in the current data. We therefore presume that adults vainly tried to integrate both sequences, leading to diminished learning under dual task conditions. This proposition might be examined in future research by inserting a condition in which the SRT sequence and the sequence of the secondary task stimuli are correlated. If our assumption is correct, adults, but not children, will learn this correlated sequence.

### Conflict of interest statement

The authors declare that the research was conducted in the absence of any commercial or financial relationships that could be construed as a potential conflict of interest.

## Footnotes

^1^More specifically, we subtracted the mean median RT (or error rate) from the median block RT (or error rate), and then divided this by the overall standard deviation of those block medians (or error rates).

^2^The data were *z*-transformed so that overall baseline differences, taking all blocks into account, were removed. However, when an analysis is performed on only part of the blocks (like is done here), group effects may still emerge.

^3^If the raw data of children and adults are analyzed separately, these results are confirmed. Sequence learning in children does not differ between the single and dual task condition, whereas learning in adults does, *F* < 1 and *F*_(1, 36)_ = 8.10, *MSE* = 227, *p* = 0.007, η^2^_*p*_ = 0.18, respectively.
