# Supplementary material for: The effect of attentional load on implicit sequence learning in children and young adults
Source: Front Psychol. 2014 May 21;5:465. doi: 10.3389/fpsyg.2014.00465 (PMC4033240; doi:10.3389/fpsyg.2014.00465)
Supplement: Supplementary file 1 [file DataSheet1.DOCX]

**Supplementary table.** Error rate percentage on secondary counting task per block per participant.

|  | **Pp.** | **Block 1** | **Block 2** | **Block 3** | **Block 4** | **Block 5** | **Block 6** | **Block 7** | **Block 8** |
| --- | --- | --- | --- | --- | --- | --- | --- | --- | --- |
| **Children** | 1 | 0.00 | 5.88 | 0.00 | 6.25 | 0.00 | 5.56 | 6.67 | 0.00 |
|  | 2 | 26.67 | 58.82 | 10.00 | 6.25 | 6.25 | 5.56 | 26.67 | 10.53 |
|  | 3 | 6.67 | 0.00 | 5.00 | 0.00 | 0.00 | 0.00 | 0.00 | 0.00 |
|  | 4 | 13.33 | 11.76 | 10.00 | 0.00 | 12.50 | 5.56 | 13.33 | 5.26 |
|  | 5 | 0.00 | 5.88 | 0.00 | 0.00 | 0.00 | 0.00 | 0.00 | 0.00 |
|  | 6 | 0.00 | 5.88 | 0.00 | 0.00 | 0.00 | 0.00 | 0.00 | 10.53 |
|  | 7 | 0.00 | 5.88 | 5.00 | 0.00 | 6.25 | 11.11 | 13.33 | 10.53 |
|  | 8 | 0.00 | 0.00 | 5.00 | 18.75 | 6.25 | 5.56 | 6.67 | 0.00 |
|  | 9 | 6.67 | 29.41 | 10.00 | 0.00 | 18.75 | 5.56 | 20.00 | 10.53 |
|  | 10 | 0.00 | 5.88 | 0.00 | 0.00 | 0.00 | 0.00 | 0.00 | 0.00 |
|  | 11 | 0.00 | 0.00 | 5.00 | 6.25 | 0.00 | 0.00 | 0.00 | 0.00 |
|  | 12 | 20.00 | 5.88 | 15.00 | 6.25 | 6.25 | 0.00 | 26.67 | 0.00 |
|  | 13 | 6.67 | 5.88 | 0.00 | 0.00 | 0.00 | 0.00 | 0.00 | 0.00 |
|  | 14 | 13.33 | 0.00 | 20.00 | 0.00 | 12.50 | 16.67 | 20.00 | 5.26 |
|  | 15 | 0.00 | 0.00 | 0.00 | 0.00 | 0.00 | 0.00 | 0.00 | 0.00 |
|  | 16 | 33.33 | 11.76 | 0.00 | 0.00 | 6.25 | 11.11 | 6.67 | 0.00 |
|  | 17 | 13.33 | 11.76 | 10.00 | 6.25 | 12.50 | 5.56 | 13.33 | 10.53 |
|  | 18 | 0.00 | 0.00 | 0.00 | 0.00 | 6.25 | 0.00 | 6.67 | 5.26 |
|  | 19 | 0.00 | 5.88 | 0.00 | 0.00 | 0.00 | 0.00 | 0.00 | 94.74 |
|  | 20 | 6.67 | 5.88 | 0.00 | 0.00 | 0.00 | 0.00 | 0.00 | 0.00 |
| **Adults** | 1 | 0.00 | 0.00 | 0.00 | 0.00 | 12.50 | 0.00 | 0.00 | 0.00 |
|  | 2 | 13.33 | 5.88 | 0.00 | 0.00 | 25.00 | 0.00 | 13.33 | 10.53 |
|  | 3 | 0.00 | 5.88 | 0.00 | 0.00 | 0.00 | 0.00 | 0.00 | 5.26 |
|  | 4 | 0.00 | 0.00 | 15.00 | 31.25 | 6.25 | 5.56 | 26.67 | 0.00 |
|  | 5 | 13.33 | 0.00 | 5.00 | 0.00 | 0.00 | 5.56 | 13.33 | 5.26 |
|  | 6 | 0.00 | 5.88 | 5.00 | 12.50 | 0.00 | 0.00 | 0.00 | 0.00 |
|  | 7 | 0.00 | 5.88 | 5.00 | 6.25 | 0.00 | 0.00 | 0.00 | 0.00 |
|  | 8 | 0.00 | 0.00 | 5.00 | 0.00 | 0.00 | 5.56 | 0.00 | 5.26 |
|  | 9 | 0.00 | 17.65 | 10.00 | 6.25 | 0.00 | 5.56 | 6.67 | 15.79 |
|  | 10 | 0.00 | 0.00 | 0.00 | 6.25 | 0.00 | 16.67 | 6.67 | 5.26 |
|  | 11 | 0.00 | 0.00 | 0.00 | 0.00 | 0.00 | 5.56 | 0.00 | 0.00 |
|  | 12 | 0.00 | 5.88 | 10.00 | 0.00 | 6.25 | 5.56 | 0.00 | 0.00 |
|  | 13 | 0.00 | 5.88 | 0.00 | 0.00 | 6.25 | 5.56 | 0.00 | 0.00 |
|  | 14 | 0.00 | 0.00 | 5.00 | 12.50 | 0.00 | 11.11 | 0.00 | 15.79 |
|  | 15 | 0.00 | 0.00 | 0.00 | 0.00 | 0.00 | 0.00 | 0.00 | 0.00 |
|  | 16 | 0.00 | 5.88 | 0.00 | 0.00 | 0.00 | 0.00 | 0.00 | 5.26 |
|  | 17 | 6.67 | 0.00 | 15.00 | 6.25 | 6.25 | 0.00 | 0.00 | 0.00 |
|  | 18 | 6.67 | 11.76 | 15.00 | 6.25 | 12.50 | 22.22 | 6.67 | 0.00 |
|  | 19 | 20.00 | 23.53 | 5.00 | 12.50 | 18.75 | 5.56 | 33.33 | 15.79 |

*Note.* Participant 2 in the children’s group and participant 19 in the adult’s group were excluded from further analyses because their mean error rate exceeded the mean error rate percentage plus 2 standard deviations. (Pp. = participant).
